# Impact of Surgical Margins in Breast Cancer After Preoperative Systemic Chemotherapy on Local Recurrence and Survival

**DOI:** 10.1245/s10434-019-08089-x

**Published:** 2019-12-23

**Authors:** K. Wimmer, M. Bolliger, Z. Bago-Horvath, G. Steger, D. Kauer-Dorner, R. Helfgott, C. Gruber, F. Moinfar, M. Mittlböck, F. Fitzal

**Affiliations:** 1 Department of Surgery, Medical University of Vienna, Vienna, Austria; 2 Breast Health Centre, Comprehensive Cancer Centre, Medical University of Vienna, Vienna, Austria; 3 Department of Pathology, Medical University of Vienna, Vienna, Austria; 4 Department of Oncology Medical, University of Vienna, Vienna, Austria; 5 Department of Radio-oncology, Medical University of Vienna, Vienna, Austria; 6 Department of Surgery, Ordensklinikum Linz/Hospital of the Sisters of Charity, Linz, Austria; 7 Department of Pathology, Ordensklinikum Linz/Hospital of the Sisters of Charity, Linz, Austria; 8 Centre for Medical Statistics, Informatics, and Intelligent Systems, Medical University of Vienna, Vienna, Austria

## Abstract

**Background:**

While “no tumour on ink” is an accepted margin width for *R*_0_ resection in primary surgery, it’s unclear if it’s oncologically safe after neoadjuvant chemotherapy (NAC). Only limited data demonstrate that surgery within new margins in cases of a pathological complete response (pCR) is safe. We therefore investigated the influence of different margins and pCR on local recurrence and survival rates after NAC.

**Methods:**

We retrospectively analysed data of 406 women with invasive breast cancer, treated with NAC and breast-conserving therapy between 1994 and 2014 in two certified Austrian breast health centres. We compared *R* ≤ 1 mm, *R* > 1 mm and RX (pCR) for local recurrence-free survival (LRFS), disease-free survival (DFS) and overall survival (OS).

**Results:**

After a median follow-up of 84.3 months, the 5-year LRFS (*R* ≤ 1 mm: 94.2%, *R* > 1 mm: 90.6%, RX: 95.0%; *p* = 0.940), the 5-year DFS (*R* ≤ 1 mm: 71.9%, *R* > 1 mm: 74.1%, RX: 87.2%; *p* = 0.245) and the 5-year OS (*R* ≤ 1 mm: 85.1%, *R* > 1 mm: 88.0%, RX: 96.4%; *p* = 0.236) did not differ significantly between narrow, wide, nor RX resections. Regarding DFS and OS, a negative nodal status reduced the hazard ratio significantly.

**Conclusion:**

There is no significant difference in LRFS, DFS and OS comparing close, wide or unknown margins after pCR. We suggest that resection in new margins after NAC is safe according to “no tumour on ink”. Resection of the clipped area in cases of pCR is emphasized.

Neoadjuvant chemotherapy (NAC) has become a cornerstone of the multidisciplinary management of patients with early, high-risk or locally advanced breast cancer (BC). It downsizes large tumours^1^^,^^2^ which may otherwise require mastectomy, thereby allowing increased breast conservation rates with equal oncological safety.^3^

Originally, a wide tumour-free resection margin was necessary to guarantee a favourable oncological outcome. In the meantime, the definition of a tumour-free margin in primarily operated patients changed to the “no tumour on ink” approach.^4^ This paradigm change is based on the debate, initiated in 2012 by Morrow et al. with the publication of “Surgical Margins in Lumpectomy for Breast Cancer—Bigger Is Not Better”.^5^ This means that the ink-stained margins must be tumour-free to declare negative margins yielding the most favourable local recurrence rate.^4^^,^^6^^,^^7^ Regarding the optimal definition for *R*_0_ resection after neoadjuvant therapy, there are insufficient data and only expert recommendations, such as that from the St. Gallen panellists, who suggest that the approach of no tumour cells in the resection margin is sufficient.^8^

Breast cancer shrinks with a scattered profile in up to 39% of patients during neoadjuvant chemotherapy.^9^ If resection with new borders shows no tumour cells (pCR) it may still be possible that scattered satellite tumour lesions might have been missed. This might explain the finding of increased local recurrence rates when chemotherapy was administered neoadjuvantly rather than in the adjuvant setting.^10^

This study aims to investigate whether narrow or wide tumour margins impact differently on the oncological outcome of patients with residual disease after NAC. Moreover, we compare oncologic outcomes of patients having residual disease and negative margins with patients having no residual disease (pCR) and thus unclear margins.

## Methods

We performed a retrospective analysis of 416 patients with BC and breast-conserving therapy (BCT) after neoadjuvant chemotherapy. Treatment was performed between 1994 and 2014 either at the Department of General Surgery of the Vienna General Hospital or at the Breast Health Centre of the Hospital of the Sisters of Charity in Linz. Before NAC, the tumour was clipped in order to facilitate identification of the primary tumour bed in case of pCR. Regimens of NAC were administered depending on the local oncologist’s choice (e.g. EC/D regimen containing epirubicin/cyclophosphamide and doxorubicin; FEC regimen with 5-fluorouracil, epirubicin, cyclophosphamide; dual combination epirubicin with docetaxel). The operation included lumpectomy and sentinel lymph node (SLN) biopsy. Breast cancer tissue was clip-marked at the time of biopsy. The day before excision, a wire was used to mark non-palpable lesions. SLNs as well as the breast tissue specimen were assessed routinely by intraoperative frozen section. The sentinel lymph node was intraoperatively identified by using blue dye. If available, dual tracer mapping by using a radiocolloid and blue dye was applied. In case of SLN metastasis, SLN biopsy was followed by level I and II axillary lymph node dissection. If resection margins were found to be involved, immediate further resection was performed. If involved margins weren’t identified in the frozen section but in the definitive histological report, a secondary operation for re-excision was mandatory. A *R*_1_ resection was defined as a surgical procedure resulting in the excision of a specimen with invasive tumour cells adjacent to one or more margin, whereas a *R*_0_ described the absence of invasive tumour cells on the inked margin of the specimen. A pathologist performed the evaluation of the margin involvement. The presence of ductal carcinoma in situ (DCIS) or lymphatic vascular invasion, even in the absence of stromal tumour invasion, was not compatible with the diagnosis of pCR. Nevertheless, the pCR referred to the lumpectomy specimen only, not to the axillary lymph node status. The majority of included patients received post-surgery radiotherapy according to local standards; in the main, conventional fractionated irradiation up to a total dose of 50 Gy with or without local boost therapy. Women who suffered from a lobular carcinoma were excluded with the intention to generate a homogenous study population. Cases where definitive histology showed a *R*_1_ resection (*n* = 17) and a mastectomy, or no further operation was performed, were excluded in order to avoid a bias regarding oncological outcome. Of the 17 patients with a *R*_1_ resection, 14 patients underwent a subsequently performed mastectomy, therefore contradicting our inclusion criteria of breast conserving therapy. Three women didn’t undergo a further operation, either for personal or for unspecified reasons. Thus, they were expected to have higher local recurrence rates and decreased survival rates. Data was extracted from a prospectively maintained patient database, histopathological reports and patients’ charts. Relevant data for this study included age, menopausal status, TNM-stage before and after NAC, preoperative biopsies including the histopathological grading, immunohistochemical hormone receptor and human epidermal growth factor receptor 2 (HER2) status—after its establishment—as well as the Ki67 labelling index, clinical stage of the tumour and the axillae, date of the operation and the final histological results. The study was approved by the local ethics committees both at the Medical University Vienna and the Hospital of the Sisters of Charity (now Ordensklinikum) Linz, Austria.

Margin widths were collected from histology reports. Negative tumour margins in patients with residual disease were grouped into close margins “*R* ≤ 1 mm” and wide margins “*R* > 1 mm”. Tumour margins in patients without invasive or non-invasive tumour cells (pCR) in the breast specimen were grouped as “RX”. Only thirteen women had a positive margin at final histology without re-excision and were excluded from the analysis.

Endpoints of this study were local recurrence-free survival (LRFS), which we defined as the time from surgery until recurrence in the ipsilateral breast as well as disease-free survival (DFS) and overall survival (OS).

### Statistical Analysis

Statistical analysis was performed by using SAS (version 9.4, SAS Institute Inc., Cary, USA). Categorical variables were described by frequencies and percentages, normally distributed continuous variables were described by mean ± standard deviation, non-normally distributed data by median with minimum and maximum. Survival curves of LRFS, DFS and OS were computed by the Kaplan–Meier method. For 5-year survival estimates, 95% confidence intervals (CI) were calculated using the asymptotic normality assumption of the log–log transformation. Group differences were compared by log-rank test and quantified by hazard ratios (HR) with corresponding 95% confidence intervals from the Cox regression model. A multiple Cox regression model was used to adjust group differences by other prognostic factors as follows: age, nodal stage after NAC (ypN stage), estrogen receptor (ER), progesterone receptor (PR), HER2 and Ki67 status, a cell proliferation index, as well as grading. ER and PR status were combined as hormone receptor (HR) status. All statistical tests were based on a two-sided significance level of 0.05.

## Results

In total, 406 patients were included in the analysis. Of those, 44% of the patients underwent surgery at the Department of Surgery at the General Hospital of Vienna, and 56% at the Breast Health Centre at the Sisters of Charity Hospital in Linz. Median patient age was 51.5 years (range 20.5–82.6 years). Median follow-up was 84.3 months (95% CI 71.6–97.1). One hundred and eighty-two (44.8%) women were pre- whereas 224 patients (55.2%) were post-menopausal. A palpable tumour at diagnosis was reported in 377 patients (92.8%). At the time of presentation, in 144 patients (35.5%) clinically lymph-node positive disease was reported, whereas 155 women (38.1%) were clinically lymph-node negative. In 107 patients (26.4%) clinical lymph node status was not documented. In 51 patients HER2 status was neither determined before nor after chemotherapy. These patients were counted as ‘nd—not done’. In the preoperative biopsy, breast cancer subtypes were represented in descending order as follows: 190 (46.8%) HR+/HER2−/nd, 118 (29.1%) HR−/HER2−/nd, 58 (14.3%) HR+/HER2+ and 40 (9.9%) HR−/HER2+ disease. All patients underwent BCT; in 48 patients a re-excision due to positive margins of the initial specimen was necessary. This represents a re-excision rate of 11.8%. Patients’ characteristics are shown in Table 1.

**Table 1:** Patients’ characteristics and an overview of the patient population

Patients’ characteristics	
Median age (years)	51.5 (range 20.5–82.6)
Median follow-up (*m*)	84.3 (95% CI 71.6–97.1)

	All patients (*n* = 406)	*R*_0_ (*n* = 358)	RX (*n* = 48)
Age			
≤ 50 years	176 (43%)	160 (45%)	16 (33%)
> 50 years	230 (57%)	198 (55%)	32 (67%)
Postmenopausal	224 (55%)	192 (54%)	32 (67%)
Palpable tumor at diagnosis	377 (93%)	334 (94%)	43 (90%)
Axilla clinically positive at diagnosis	144 (35%)	126 (35%)	18 (38%)
Tumor biology			
HR+/HER2−/nd	190 (47%)	183 (39%)	7 (15%)
HR−/HER2−/nd	118 (29%)	94 (26%)	24 (50%)
HR+/HER2+	58 (14%)	50 (14%)	8 (17%)
HR−/HER2+	40 (10%)	31 (9%)	9 (19%)
Grading			
I/II/X	200 (49%)	182 (51%)	18 (38%)
III	206 (51%)	176 (49%)	30 (63%)
Ki67-Status			
≤ 20	92 (23%)	92 (26%)	0 (0%)
> 20	137 (34%)	114 (32%)	23 (48%)
X/nd.	177 (44%)	152 (42%)	25 (52%)
Stage			
ypT0/X	50 (12%)	2 (1%)	48 (100%)
ypTis	30 (7%)	30 (8%)	**–**
ypT1	210 (52%)	210 (59%)	**–**
ypT2	102 (25%)	102 (28%)	**–**
ypT3/4	14 (3%)	14 (4%)	**–**
Re-resection performed	48 (12%)	46 (13%)	2 (4%)
Postoperative RT	389 (96%)	343 (96%)	46 (96%)
Local recurrence	39 (10%)	36 (10%)	3 (6%)
Contralateral recurrence	13 (3%)	11 (3%)	2 (4%)
Axillary recurrence	9 (2%)	9 (3%)	0 (0%)
Distant metastasis	86 (21%)	83 (23%)	3 (6%)

*CI* confidence interval, *y* years, *m* months, *HR* hormone receptor, *HER2* human epidermal growth factor receptor 2, *RT* radiotherapy, +positive, − negative, *nd* not done

A margin width of *R* ≤ 1 mm was reported in 74 patients (18.2%), *R* > 1 mm in 284 patients (70.0%) and RX due to pCR in 48 patients (11.8%). During follow-up 64 patients died (15.8%). Local recurrence was diagnosed in 39 patients (9.6%) whereas 13 patients (3.2%) suffered from contralateral recurrence. Therefore, in 354 cases (87.2%) no disease recurrence was observed. Axillary recurrence was found in 9 cases (2.2%) whereas distant metastasis was reported in 86 patients (21.2%).

The definitive histological report showed ypT0/X in 50 patients (12.3%), ypT1 in 210 (51.7%), ypT2 in 102 (25.1%), ypT3/4 in 14 (3.5%) and ypTis in 30 (7.4%). Positive nodal status (ypN+) was observed in 175 patients (43.2%). ypN- was found in 230 patients (56.8%). In one patient the lymph node status was not available. In one case ypT0/X was classified by pathologists although lymphangiosis was found in the tumour bed. This patient was classified as “*R*_0_”, because the existence of residual disease was not considered as pCR.

Postoperative whole breast irradiation with or without additional local boost therapy was administered in 389 of 406 patients (95.8%). Seventeen patients (4.2%) did not receive radiotherapy due to individual decisions: either patients’ or interdisciplinary tumour board decisions.

### Oncologic Outcome

**Fig. 1:**
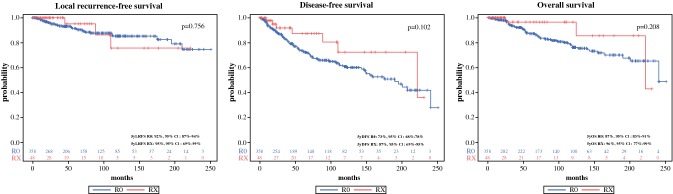
Survival curves “*R*_0_” versus “RX”. Kaplan–Meier curves show LRFS, DFS and OS for patients with either “*R*_0_” (red solid) or “RX” (blue solid) resections

Regarding LRFS as well as OS, no significant difference was found for either margins described as “*R*_0_” or “RX” (5yLRFS for RX: 95.0%; 95% CI 69.5–99.3 vs. *R*_0_: 91.5%; 95% CI 87.4–94.3; *p* = 0.756; 5yOS: RX: 96.4%; 95% CI 77.2–99.5 vs. *R*_0_: 87.3%; 95% CI 82.6–90.7; *p* = 0.208). There was a non significant trend of a higher 5-year overall survival in patients with Rx with an absolute amount of 9.1%. A trend towards a better DFS, yet not significant, was seen in women after RX margins when compared to *R*_0_ margins (5yDFS: RX: 87.2%; 95% CI 0.68.5–0.95.2 vs. *R*_0_: 73.5%; 95% CI 67.8–78.3; *p* = 0.102). Survival curves are shown in Fig. 1.

**Fig. 2:**
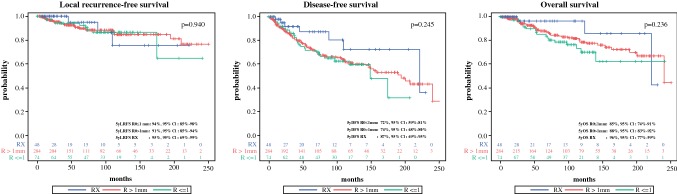
Survival Curves “*R* ≤ 1 mm”, “*R* > 1 mm” and “RX”. Kaplan–Meier curves show LRFS, DFS and OS for patients with “*R* ≤ 1 mm” (green solid, “*R* > 1 mm” (red solid) or “RX” (blue solid) resection

Even after further dividing of *R*_0_ resections into “*R* ≤ 1 mm” and “*R* > 1 mm”, no significant difference was found for either LRFS (5yLRFS: *R* ≤ 1 mm: 94.2%; 95% CI 85.3–97.8 vs. *R* > 1 mm: 90.6%; 95% CI 85.5–94.0 vs. RX: 95.0%; 95% CI 69.5–99.3; *p* = 0.940) or for DFS (5yDFS: *R* ≤ 1 mm: 71.9%; 95% CI 59.4–81.1 vs. *R* > 1 mm: 74.1%; 95% CI 67.5–79.5 vs. RX: 87.2%; 95% CI 68.5–95.2; *p* = 0.245) or for OS (5yOS: *R* ≤ 1 mm: 85.1%; 95% CI 74.0–91.7 vs. *R* > 1 mm: 88.0%; 95% CI 82.5–91.8 vs. RX: 96.4%; 95% CI 77.2–99.5; *p* = 0.236). Survival curves for those three groups are shown in Fig. 2.

**Fig. 3:**
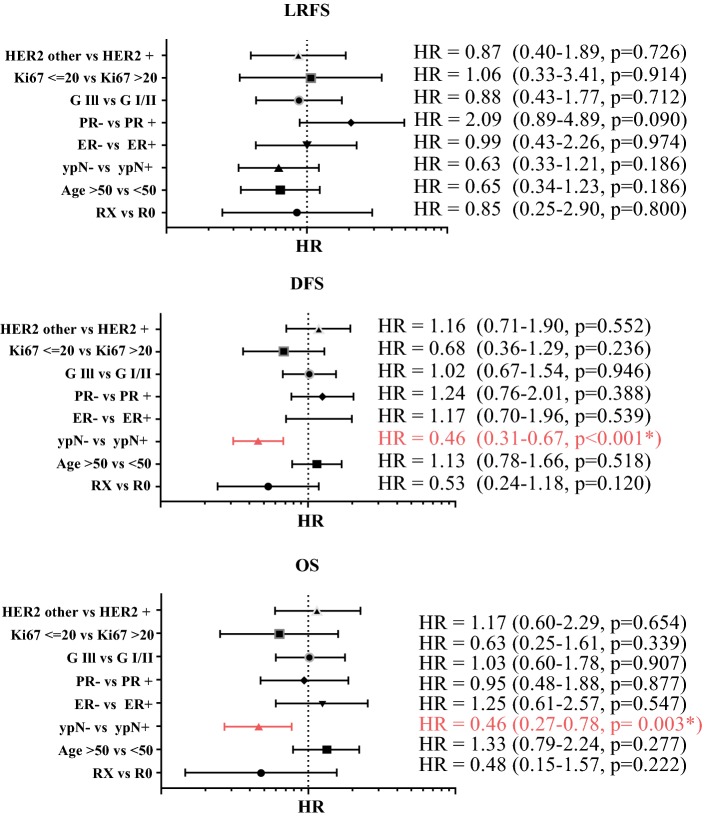
Multivariate analysis of risk factors. Multivariate analysis of risk factors shows that a negative nodal status after NAC significantly lowered the HR for events regarding OS and DFS. *HER2* human epidermal growth factor receptor 2, *Ki67* proliferation index, *G* grading, *PR* progesterone receptor, *ER* estrogen receptor, *ypN* N stage after neoadjuvant chemotherapy, *R* margin status, statistically significant results are in *red*

In a multivariate analysis after adjusting the proportional hazards regression analysis for age, ypN stage, ER, PR, HER2 and Ki67 status and grading, it became apparent that a negative nodal status was independently prognostic for an improved OS (HR = 0.46, *p* = 0.004, 95% CI 0.27–0.78) and DFS (HR = 0.46, *p* < 0.0001, 95% CI 0.31–0.67) but not for LRFS (HR = 0.63, *p* = 0.164, 95% CI 0.33–1.21) (Fig. 3).

## Discussion

We report a large retrospective data analysis of 406 women in two different breast health care centres in Austria treated with NAC and breast conserving surgery. Our data show that simple lumpectomy within new boundaries and resecting the clipped tumour bed followed by radiotherapy is safe. In our population, the definition “no tumour on ink” yields excellent LR rates after 5 years of 8.5%. In women after pCR only, 4.5% had a LR after 5 years. There was no difference in LR rates, either in patients with close or with wide negative margins. In women with pCR who underwent resection of the clipped tumour bed within the new boundaries, the same LR rates were found.

Intratumoural heterogeneity causes heterogeneous response to NAC that further leads to unpredictable tumour shrinkage.^11^^,^^12^ While concentric tumour shrinkage during NAC leads to a solitary residual tumour, other patterns of shrinkage can result in multifocal or patch-like lesions, or even a residual tumour with satellite lesions.^9^ Residual patchy lesions after NAC impede the assessment of the required resection extent. As a consequence, a higher incidence of positive margins after NAC can be assumed. Therefore, Volders et al. compared the incidence of positive margins in primary operated patients and in patients undergoing NAC. This Dutch nationwide pathology study was conducted by analysing core biopsies, operation specimens and re-excision as well as mastectomy specimens. They found a 3 times higher risk of *R*_1_ resections after NAC (OR 2.94, incidence: 24.3% vs. 10.2%).^13^ This might be an explanation for the higher rates of local recurrence in patients receiving NAC. The Early Breast Cancer Trialists’ Collaborative Group (EBCTCG) demonstrated in a meta-analysis that the same chemotherapy given preoperatively correlates with higher local recurrence rates than when given postoperatively. A 15-year local recurrence rate of 21.4% was observed in the NAC group, whereas it was 15.9% in the adjuvant chemotherapy group (*p* = 0.0001).^10^

Given that NAC can lead to higher rates of *R*_1_ resections, the influence of positive margins on the oncological outcome should be considered. Gentilini et al. focused on the difference in local recurrence rates between *R*_0_ and *R*_1_ resections. They found that positive margins have a marginally significant higher incidence of local recurrence after 3 years than negative margins (13.3% vs. 4.7%, *p* = 0.05).^14^ In our study, *R*_1_ resections were excluded; consequently, a comparison with our findings cannot be done. Nevertheless, the optimal margin width remains unclear in the neoadjuvant setting.

Therefore, the primary objective of our trial was to answer the question as to whether the “no tumour on ink” approach is safely applicable in the neoadjuvant setting. We primarily focused on the difference of oncological outcome parameters in patients with wide and narrow specimen margins.

As we could show that narrow resections margins (*R* ≤ 1 mm) do not lead to a statistically significant difference in local recurrence-free, overall, or disease-free survival in comparison to wider resections (*R* > 1 mm), we can confirm that “no tumour on ink” is reliable and safe after NAC. Nevertheless, a trend towards improved survival of patients with RX resections was observed in Kaplan–Meier curves.

Choi et al., who recently published the results of a retrospective analysis, came to a similar conclusion. In patients after NAC, no differences in 5-year LRFS, OS or DFS could be found between patients with either closer (< 2 mm) or wider (≥ 2 mm) margins.^15^

Likewise, Tyler et al. investigated whether close margins (< 2 mm) have an impact on patient outcomes. In a study cohort of over 10,000 patients and a median follow-up of 8 years, where 87% of patients were administered systemic chemotherapy, close margins were not associated with increased local recurrence or decreased breast cancer specific survival. In conclusion, they suggest that omitting re-excisions of positive or close margins might be an acceptable approach in selected cases scheduled for adjuvant radiotherapy.^16^ Nevertheless, it must be that they didn’t distinguish between neoadjuvant and adjuvant chemotherapy.

A further aim of our study was to clarify whether pCR influences the oncological outcome.

Pathologic complete response in general is associated with improved survival.^17^^,^^18^ Nevertheless, the definition of pCR is still under discussion, as it remains unclear whether the presence of ductal carcinoma in situ (DCIS) is compatible with the term pCR.^19^^–^21

In our study cohort, we determined that residual DCIS in the operation specimen didn’t match the criteria of pCR. Applying this, neoadjuvant chemotherapy led to pCR in 12.3% of our patients. Our study might have been underpowered to correlate pCR with improved oncological outcome, as LRFS, OS and DFS didn’t differ significantly when compared to patients with *R*_0_ resection. Nevertheless, we also observed a noticeable trend towards improved DFS (5yDFS: RX: 87.2% vs. *R*_0_: 73.5%; *p* = 0.102). Taken together, in cases of pCR, a resection of the clipped tumour bed within the new boundaries is safe as well.

In the presented study population, we observed a re-operation rate due to positive margins after the first breast conservation attempt of 11.8%. An earlier study of our patients without neoadjuvant therapy demonstrated a 9% re-resection rate, suggesting no significant difference in re-resection rate with or without neoadjuvant treatment.^22^

In an adjusted Cox model we found that a negative nodal status after NAC was independently correlated with the HR for events regarding OS and DFS. This finding is supported by a study conducted by Van Nijnatten et al., who observed that ypN1-3 was significantly associated with worse prognosis.^23^ In our cohort, we couldn’t find any influence of tumour biology (measured by ER, PR, HER2 receptor status, Ki67, grading), age or pCR on outcome. In contrast, Galvez et al. found that ER+, PR+, luminal A-disease, cN0 as well as pCR were associated with improved OS and DFS.^24^ In this regard, further investigations, focusing on post-neoadjuvant therapy options are warranted.

Our study has some limitations, which must be taken into consideration when interpreting our findings. In particular, the retrospective as well as the non-randomized design must be mentioned. Furthermore, the small number of patients with “RX” in their final pathologic report might weaken the conclusion in this subgroup. However, it must not be assumed that these limitations influence the validity of the results presented here regarding close or wide margins an their impact on local recurrence and survival rates. In some cases, data regarding HER2 (in 51 patients) and Ki67 status (in 177 patients) could not be obtained, as several women were treated at times when HER2 and Ki67 immunohistochemistry was not yet routinely performed. Therefore, a detailed subgroup analysis of the impact of tumour subtypes on outcome is restricted. Detailed documentation on adjuvant radiotherapy regimes including local boost therapy is not available.

Taken together, large, randomized controlled trials investigating the oncological outcome of patients undergoing NAC followed by BCT would be desirable and necessary to confirm our findings.

In conclusion, the data presented here suggests that there is no significant difference in oncological outcome, measured by LRFS, DFS and OS, between narrow or wider margins or RX resections due to pCR. This implies that breast conserving surgery after neoadjuvant chemotherapy is oncologically safe even if the definition of *R*_0_ resections as “no tumour on ink” is applied.
